# Woman, Mother, Wet Nurse: Engine of Child Health Promotion in the Spanish Monarchy (1850–1910)

**DOI:** 10.3390/ijerph17239005

**Published:** 2020-12-03

**Authors:** José Siles-González, Laura Romera-Álvarez, Mercedes Dios-Aguado, Mª. Idioia Ugarte-Gurrutxaga, Sagrario Gómez-Cantarino

**Affiliations:** 1 Department of Nursing, University of Alicante, Carretera de San Vicente del Raspeig s/n, 03690 Alicante, Spain; jose.siles@ua.es; 2 Department of Nursing, Physiotherapy and Occupational Therapy, University of Castilla-La Mancha, 45071 Toledo, Spain; Maria.Ugarte@uclm.es (MI.U.-G.); sagrario.gomez@uclm.es (S.G.-C.); 3 Yepes Health Center, Health Service of Castilla-La Mancha, Av. Santa Reliquia 26, 45313 Toledo, Spain; mded@sescam.jccm.es

**Keywords:** infant care, health promotion, breastfeeding

## Abstract

In Spain, the wet nurse increased the survival of children through care and breastfeeding of other women’s children. They had a great development together with the Spanish monarchy between 1850 and 1910. The aim is to identify the role of wet nurses in the Spanish monarchy and the survival of the royal infants (s. XIX–XX). A scoping review is presented to study documents about the wet nurse in the Spanish monarchy. Applying the dialectical structural model of care (DSMC). Recognizing five thematic blocks that shape the historical-cultural model. Books, decrees and databases were analyzed: Scopus, Scielo, Dialnet, Cuiden, Medline/Pubmed, CINAHL, Science Direct and Google Scholar, from January to July 2020. The selection process was rigorous because it was difficult to choose. They had to overcome medical and moral exams. The selected rural northern wet nurses emigrated to Madrid. The contract was regulated by laws and paid. Wet nurses were hired by the monarchy due to health problems of the biological mother and a need for greater offspring. The wet nurse wore a typical costume, a symbol of wealth. The northern wet nurses hired by the monarchists have been the engine that has promoted the health of infants through the breastfeeding process.

## 1. Introduction

In Spain, the term “*ama de leche*” and “*ama de cría*” have been used throughout history to refer to the wet nurse. The “*ama de leche*” is the woman whose sole function is to breastfeed the newborn. On the other hand, the “*ama de cría*” also performs other functions such as education and care of the child [1]. The etymological origin of nurse comes from “*nutricia*”, referring to the salary that these women received for their profession [2]. However, the term “wet nurse” is defined by the Royal Spanish Academy as “a woman who breastfeeds a foreign child” [3]. This woman breastfed every 3 to 4 h, in addition to other functions such as entertaining, laying down, or grooming the newborn [4]. The relevance of this study is justified for three main reasons. On one hand, by the need of finding out the role wet nurses played in reducing infant mortality in Spain, not only in the royal court but also in the sectors of society which imitated these practices and incorporated them into their lives. On the other hand, it is justified by the assessment of the importance of monarchs in the selection and promotion of wet nurses inside and outside the court. Finally, this review can be useful in the further study of the similarities and differences that existed between the royal wet nurses of the different European monarchies during the period studied. This profession can be considered as one of the first jobs performed by women since ancient times [5]. In fact, this figure already appears in the Ebers Papyrus (1550 BC) and in Babylonian codes, such as Hammurabi and the Laws of Esnunna [5,6,7]. This trade was socially known in Ancient Greece and Rome, where wet nurses administered their contracts in market areas known as *lactarias* [8,9].

The regulation of wet nurses appears early in Spain, undoubtedly due to the urgent need to alleviate infant mortality and specifically infanticide. Since the 12th century, the nurse has been closely associated with the Spanish monarchy. This transformation of female biological activity, so essential for society, already appears in the Royal Charter of Castilla (drawn up between 1252 and 1255), where, to reinforce local law, the abandonment of children was punished, and the death penalty was sentenced to who let an infant die for not having taken him to raise [10]. The “Partidas” enlightened parents on how to raise their children, and in the newest compilation, the way to proceed in cases of the orphanage was prevented. Even the general charity regulations of 27 December 1821 established the need to open a maternity home for breastfeeding in each province, with the aim of saving children by avoiding infanticides and preserving the honor of the mothers (Article 42). This Regulation established the work periods of the wet nurses, adjusting to the physiology of the child: the wet nurses breastfeed the children during the first two years, a period in which the rearing period began (Articles 58–61) [10,11]. One year later, a law of 23 January 1822 (Title III, Articles 41 and 50–70) addressed the regulation of the living conditions of orphaned children in charity establishments. Subsequently, the law of 20 June 1848 and the subsequent regulations dealt with the protection of foundlings, orphans and the homeless [12]. The protection of children at the political and legislative level will lead during the fifties and sixties of the 19th century, to the creation of the “maternity and foundling houses”. These places were consolidated and, the figure of the wet nurse took strength and responsibility in the survival of children in Spain. These women fed the most disadvantaged infants, who belonged to social classes with fewer economic resources. These children did not have a mother, either due to death or abandonment. This is why a series of provincial infrastructures linked to city councils and county councils were created. As an example of this, in Alicante (Spain), the regulation of 9 March 1862 made explicit the general conditions that external wet nurses must have [10,11,12,13]. The Law of 12 August 1904 extended its protection to all children under the age of ten, and the Superior Council for Children with its corresponding provincial and local boards was created as a regulatory body [14]. The regulations for this law were promulgated on 24 January 1908 [15]. Such abundant regulation, together with the copious documentation on wet nurses in the form of payroll and subsidies (municipal and provincial), constituted evidence that cannot be ignored: wet nurses were an industry that was essential to alleviate the high rates of infant mortality [10] (Table 1).

It should be considered that during the years 1901–1905, Spain had an infant mortality rate of 172.8 per thousand, and this was the highest of all the countries in Europe, except for Portugal [11]. For this reason, during the 20th century in Spain, some children were fed with another woman’s milk in an unselfish way when the biological mother could not breastfeed her newborn. It was called altruistic breastfeeding. On multiple occasions, women in urban areas, after giving birth, began to work in factories, abandoning their home and their child. These were breastfed by another wet nurse with a lower salary, or ultimately by another mother in the family [10,16].

Women from northern Spain who were hired by the monarchy improved their quality of life. Since in their place of origin, they had fewer resources and less purchasing power. However, when hired by the monarchy, this vital situation changed. They acquired economic privileges, food resources, better housing, even clothes for herself, her children and her husband [12]. After the lactation and rearing periods, they were linked to them as high-level maids. This figure acquired an important role in the Spanish monarchy, considering the palace wet nurses as the most fortunate given the condition of their “milk children” [12,13]. Therefore, different groups of wet nurses are historically identified during the 19th century in Spain. On one hand, in the private context, the figure of the nurse who was advertised in the newspapers offering his services to middle-class families was worth highlighting, even to a minor extent. These women had a lower salary than the monarchy wet nurses, and they did not own luxury clothes. They had an improvement in her quality of life due to obtaining resources and money. Others offered their services to the lower-class neighborhood population. These women had worse conditions, either salary, food and home resources and fees without forgetting the work of the wet nurses in the foundling houses, where the abandoned children lived. The work done by these women was paid for by the local government [13].

Due to the characteristics of northern women, the Spanish monarchy had a special interest in them. These young mothers began to be in demand, especially those from Valle del Pas, who migrated to urban areas, such as Madrid [17]. These women were considered healthier and more right to perform this job due to their robust physical state and their life in nature [18]. For this reason, the royal medical offices sent to carry out the selection process established strict physical and moral requirements to be met by these women. In this way, the responsibility of choosing the nurse fell on the surgeons sent by royalty to the rural areas of northern Spain [19]. This medical cabinet selected the wet nurses one month before the stipulated date of childbirth [14]. These women took care of housework after the breastfeeding period. This work of “maids” was socially accepted since the role of a woman at that time was situated in a religious setting in a convent, as a wife or as a housewife performing domestic tasks in raising and caring for children [14,19].

In the following sections, first, the methodology used to carry out the analysis is presented in accordance with the research objectives. The main objective is to relate the figure of the wet nurse of the Spanish royalty with the survival of infants during the 19th and early 20th centuries. The secondary objectives are: (1) to describe the characteristics of wet nurses in the royalty; (2) analyze the geographical location of these women; (3) know the legislation and regulation of the profession. The results section identifies the most significant findings found after the review was carried out. Next, a discussion is presented, where the results are compared with the previous knowledge of the objective of the study. Finally, the main conclusions, limitations and future lines of research are shown.

## 2. Materials and Methods

### 2.1. Study Design

In this article, a scoping review was carried out as a method to address the objective of the study. The aim is to relate the work of the wet nurse in the Spanish monarchy with the survival of the royal infants during the 19th and 20th centuries. These reviews are an ideal tool for determining the scope of a set of publications on a specific topic. They give you an idea of the volume of publications and studies available. In addition to acquiring a vision of the focus of the documents, which allows researchers to an evaluation, synthesis, and critique the evidence inherent to the objective of the study [20,21]. The dialectical structural model of care (DSMC) [22] was used due to its ability to delve into the cultural and social roots of structures especially linked to the sexual and gender division of labor. This model identifies the functional dynamics of the structures allowing the analysis of the causes that produce their changes.

**Figure 1:**
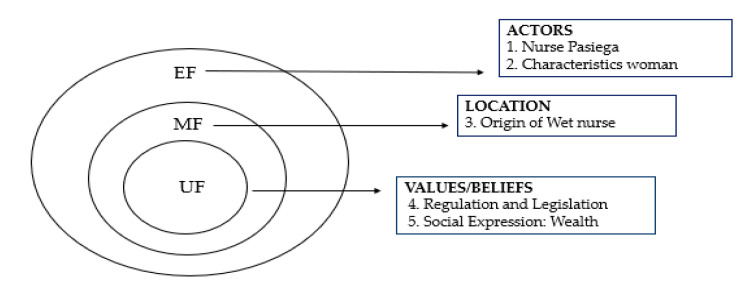
Theoretical dialectical structural model of care (DSMC) model: application of its structures. Source: authors’ own elaboration.

The DSMC methodology is based on structures that serve as support for the data management and analysis process. In this research, its application is important due to the social, cultural and care study in which we enter. Therefore, the structures that apply to it are (1) functional unit (UF), which includes norms, values, beliefs, knowledge and feelings that give rise to social systems and that determine the gender and the sexual division of work, so decisive in the emergence of these professions where the physiological merges with the work; (2) functional framework (MF), related to the place where activities are carried out; (3) functional element (EF), includes social actors, responsible for managing care. These constitute an adequate tool for the ordering and analysis of data to obtain a global view of the historical phenomena from the perspective of cultural history [23]. Within this research, five thematic blocks were developed focused on the DSMC, and each one of them encompassed the structures that shape this historical and cultural model [24] (Figure 1).

### 2.2. Search Strategy

The scope review process begins with an exploratory research question [25] aimed, in this case, at systematically synthesizing and critiquing existing knowledge [26]. In this case: “what was the link between the nurses and the Spanish monarchy during the 19th and early 20th centuries?” This review included several steps [27].

Initially, the topic was identified wet nurse to the Spanish royals, and a research question was established: how did the work of the wet nurses influence the survival of the royal infants? The study was set within a historical period (1850–1910) and the care culture of the time. The dialectical structural model of care (DSMC) was applied. The researchers agreed on eligibility criteria, which should contain information on the figure of the Spanish royal nurse and child survival. The review includes documents describing the origin, selection process and privileges of the royal wet nurses. The review also includes studies containing the selection process of wet nurses and the salary. Studies on the origin of the royal wet nurse, legislation and disguise were selected. The review includes articles, dissertations, conference proceedings and peer-reviewed reports. However, it excludes conference abstracts, proposals and editorials. The articles included were published after 1850. They were also to be found in English, Spanish and Portuguese. There are no restrictions on the place of work of wet nurses (foundling houses, private and royal wet nurses), the type of article or the country of publication.

Subsequently, a search for documents was carried out from January to July 2020. Libraries of the University of Castilla-La Mancha, the National Library of Spain, the Public Library of Toledo, as well as the Historical Archive of Toledo and Alicante (Spain) were consulted. On one hand, several sources were consulted: (1) Bibliographic Database on Health in Latin America (CUIDEN); (2) Medline/PubMed; (3) Scopus; (4) Web of Science; (5) Scielo; Science Direct and (6) Google Scholar. MeSH and DeSH terms were used to carry out a more exhaustive and advanced investigation starting from the Boolean operator “[AND]”, “[AND]”, “[OR]”. Additionally, word combinations were used, if applicable, to reflect the syntax and search rules common to individual databases. The descriptors were the following: infant care, intercultural care, health promotion, breastfeeding, historical research, care, society, infant mortality, and child survival. After searching, eliminating, and selecting the articles, the selected ones were coded and identified (Table 2).

### 2.3. Review Process

This scoping review was conducted from January to June 2020. This type of review aims to map the evidence that supports a particular area of research and to identify gaps in existing evidence. This methodology does not aim to analyze the methodological quality of the studies included or to find the best scientific evidence, but to map the existing scientific evidence. By means of the consensus of the authors (J.S.-G., L.R.-A., M.D.-A., M.I.U.G. and S.G.-C.), articles, books, dictionaries, documentation of historical archives and laws were reviewed. A figure of 66 documents was reached, which met the requirements reflected in the inclusion and exclusion criteria. Note that royal decrees, norms as well as legal provisions were consulted, presented in Table 1, with the intention of specifying and listing part of their most relevant articles in relation to the subject of study.

### 2.4. Data Analysis

The content analysis of the documentation was carried out from a qualitative perspective through an objective and systematic [24]. The steps that were carried out for the analysis consisted of: (1) a thematic link; (2) a preliminary classification of documents based on content and organizational criteria; (3) a selection and extraction of relevant information, according to the scope review criteria, in order to allow results and conclusions [24,25,26]. The selected articles were analyzed from the point of view of the five thematic blocks studied, each one of them encompassed within the structures that make up the DSMC (dialectical structural model of care): (1) the appearance of the breeder Pasiega; (2) physical, sanitary and moral characteristics of the northern wet nurses; (3) provenance of the royal nurse; (4) legislation and regulation of wet nurse work and, (5) wet nurse dress. All these blocks were contextualized within the prevailing social reality in Spain during the study period (1850–1910).

In order to extract and summarize the data, in this historical investigation, the researchers performed an inferential interpretation. An attempt was made to know the reality already investigated and written, facing the social and health environment of the time studied in Spain. The first and second authors (J.S.-G. and L.R.-A.) performed general data extraction. While the third author (M.D.-A.) examined, the findings found. The fourth and fifth authors (I.U.-G. and S.G.-C.) identified the common thematic lines, which are included within the structures that shape the DSMC, from its nucleus (UF), middle zone (MF) and its external part (EF). When there was a discrepancy in the choice and inclusion of studies, it was resolved by consensus among the investigators (Table 3). Studies chosen for analysis based on the degree of rigor were not excluded since the objective of the scoping review was to synthesize the results of the reviewed research, to extrapolate greater knowledge and vision of health care to the scientific world [24]. Thus, after reading and rereading the chosen articles, it was possible to answer the guiding question proposed in this study: “What was the link between the wet nurses and the Spanish monarchy during the 19th and early 20th centuries?”: “what was the link between the wet nurses and the Spanish monarchy during the 19th and early 20th centuries?”

## 3. Results

At the end of the 19th century and the beginning of the 20th, in Spain, the wet nurse was in great demand for medical, social, and political reasons. Spain had an infant mortality rate of 172.8 per thousand, the highest of all European countries. It should be noted that infant mortality was not only high in Spain, as the number of deaths was also high in much of Europe and even North America. At the same time, it is true that newborns of poor mothers or from disadvantaged socioeconomic families had worse hygienic and nutritional conditions, which meant a high risk of child infection, and therefore, of death [28]. These high numbers worried the Spanish population, but especially the monarchy, due to the need to have offspring able to survive. In a politically unstable period, where the alternation between liberals and conservatives was latent in Spain, the social, political and health situation was in crisis [29]. For this reason, one of the solutions to the mortality of newborns and of their infants, proposed by the monarchs, was to hire wet nurses. However, the royalty wanted to make sure that the chosen woman was the most suitable for these functions since she had to breastfeed her infant with the best possible quality of milk so that the newborn could survive and reign in the future [30].

### 3.1. The Rise of the Northern Wet Nurses

The validity of a series of ideal traits of the wet nurses, at that time, is the consequence of a process of social construction that determines the “functional element” (EF). In this way, in this study period (1850–1910), the great rise of the wet nurse began in Spain from Cantabria, Asturias, Navarra, the Basque Country and Burgos [17]. Occasionally, some women were originally from La Mancha and Segovia [28]. It can be commented that there were several wet nurses who suckled the Spanish royalty. However, those that were not selected in the first place as an official breeding wet nurse remained in La Pajarera (Madrid palace) so that in case of need, they could replace the chosen wet nurse due to the high quality of the milk of these women [9].

It is worth highlighting Francisca Ramón de Peñacastillo, a 21-year-old young woman who breastfed Queen Elizabeth II. Furthermore, María Gómez raised Alfonso XII, the son of this sovereign, with her milk [31] (Table 4). The lactation period lasted approximately two years, and after its completion, the fate of the wet nurse was to return to her area of origin or to stay in the workplace as an “*ama seca*” or “*ama de brazos*”, both in care of the children, improving their survival. These women were not just employees, but they became a member of the family. This affective bonding transformed the wet nurse’s work activity e into a relationship that went beyond the pure venality and that integrate the wet nurse into the nucleus establishing parental relationships that equidistant to the siblings of milk with blood, in accordance with the values about the family [25,32,33].

Due to the privileges that the wet nurses acquired, such as being considered members of royalty, the choice of these women was strict. This process began with the contact of the monarchy with the town councils and districts, which oversaw notifying the future wet nurses that, by providing a residence card, they could appear for the selection exams [33]. Physical and moral characteristics were analyzed by royal doctors and parish priests from the woman’s area of origin, respectively. The doctors in charge of the physical examinations once carried out wrote a report that reflected the good health of the wet nurse, her son and even her husband [34,35]. The women eligible for the exam obtained a notebook, where the contracts and dismissals of the different families in which they worked were sealed. Therefore, the possession of the passbook was mandatory to perform this job [9,36].

### 3.2. Physical and Moral Characteristics of Northern Wet Nurses

In the EF, the physical characteristics and quality of the milk are integrated as the model profile of these women dedicated to breastfeeding [24,25]. In the first examination, the woman was assessed in various aptitudes. On one hand, they had to have a chest without respiratory problems and being vigorous, but not excessively, to avoid problems in lactation and malnutrition in the newborn that increased infant mortality [37]. On the other hand, their physical features were observed since these were linked to diseases such as ringworm, which mainly affects children, such as their hair, which should be strong and not have bald spots [35]. In addition, red hair, associated with witchcraft [38], was excluded. Regarding the buccal region, the mucous membranes and breath were especially observed [39]. Those who suffered from halitosis, absence of teeth, cavities or had gum problems were excluded since oral alterations were associated with a rejection of the baby towards the breastfeeding and therefore a worse digestive system, which would lead to an increase in mortality of these children [18,35,40]. The eyes were also considered in the face, and even the gaze, since it had to be pretty, excluding cross-eyes, a purely esthetic issue [41].

These wet nurses were required to be vaccinated to avoid contagious diseases and to prevent their transmission to newborns [30]. In addition, the genitalia and anus were examined. Those with sexually transmitted infections, such as syphilis, which is transmitted through breast milk, causing the death of newborns, were excluded. The absence of skin disorders was a requirement that these women had to meet, and their brown color was even positively valued since they were considered healthier [18,32]. On the other hand, epilepsy problems, a poorly known disease, were excluded because they could affect breastfeeding and the survival of these children [32].

In the physical examination, the most valued area of the body was the chest. Great attention was paid to the volume and the adipose tissue of the mammary gland [1,9]. The ideal was a chest of a medium volume and better with less fat. The nipple should not be bulky or short, as it would make the sucking difficult for the newborn, producing malnutrition and, therefore, an increase in infant mortality [18,28]. It was even important how the milk left the breast since it had to do this at least through ten or twelve orifices to avoid a deficit and a state of malnutrition in the newborn that could harm their health, even be fatal [28,32]. On the other hand, the milk was considered of good quality if when you put a drop in the eye, it did not irritate it, if when mixing the milk with the water it dissolved easily, and even if it did not bubble when pouring milk in an alkaline or acid medium. This milk should be rich in lactose, sweet, odorless, buttery, uniform and bluish-white. The density of the milk was checked by putting a drop on the nail, which should not slip or stick [9]. Finally, lactoscopes and the Beranger scale [40] began to be used. The lactoscope was used to determine the proportion of cream in the milk in relation to the opacity of the milk. It is formed by two glass plates, parallel, between which the milk is placed, moving them away or closer and measuring their transparency on a scale located on the support of the mobile lens. It is based on the idea that the milk fat globules are opaque and surrounded by a much more transparent liquid. As the amount of fat increases, its transparency will decrease. The Beranger scale was made of wood, marble, iron and brass. It was used to weigh the milk [42].

Another aspect that was addressed in the physical examination was age. The appropriate range was between 19 and 26 years, although it was extended to 35, with the aim of increasing the birth rate and reducing infant mortality. Another possible argument was that, as they got older, the probability that they were new mothers decreased, as they were seen as inexperienced and less emotionally strong. This problem could affect the quality of the milk and, consequently, the survival of these children [33,43].

**Figure 2:**
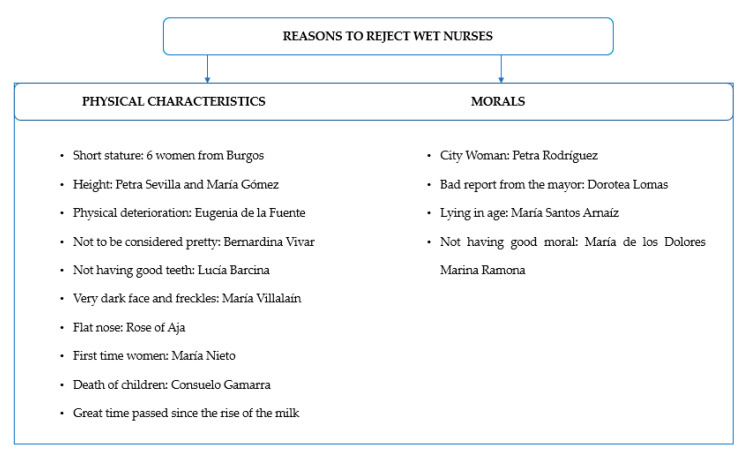
Reasons to reject wet nurses. Source: authors’ own elaboration.

Regarding the second exam, the morality one, it was carried out by a priest, who had to testify, by means of a certificate, that both the wet nurse and her family had Christian morality, correct values and customs (functional unit of the historical context that delimited this type of activities so closely linked to gender and the sexual division of labor) [25] (Figure 2). At the time of the study, it was believed that these values passed from the wet nurse to the newborn through the milk [44]. In addition, the wet nurse needed another authorization from the husband to practice the job [32].

The benefits of the profession were not exclusively for the wet nurse but involved the whole family. Even the wet nurse’s children were considered as “milk brothers” of the royal infant, having received breastfeeding from the same woman. This fact significantly increased the survival and the birth rate of the wet nurse’s family, as it improved the family’s economic situation [45,46]. The retribution of these women was in the form of salary, together with food, clothing, and even a job for her son [10]. Although it is true that between 1858 and 1868, the average salary of these women was around 60 or 80 reales, while in 1904, the amount was 40 pesetas per month [44].

### 3.3. Origin of the Monarchical Wet Nurse

The functional framework (MF) enables the presentation of the results regarding the origin of wet nurses. Specifically, in the 19th century (1830), King Ferdinand VII sent an expedition to Cantabria, which was led by Asso and Merino (doctor–surgeon), who had to choose a nurse for his future lineage [19,33,40].

**Figure 3:**
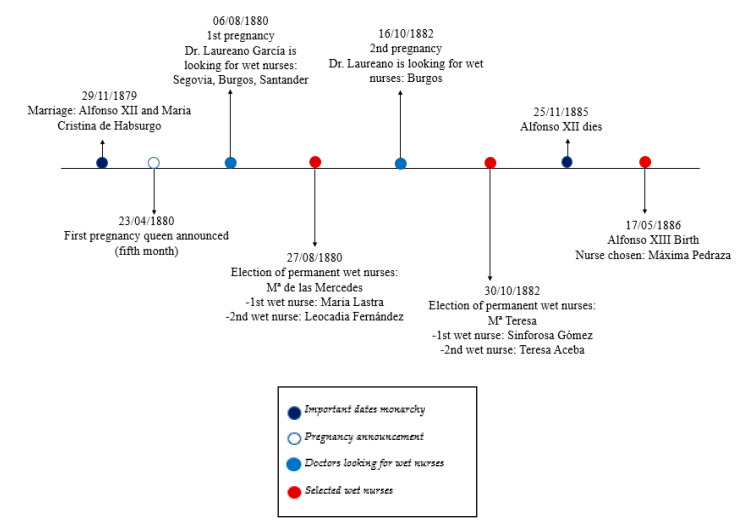
Raising of infants by wet nurses during the reign of Alfonso XII. Source: authors’ own elaboration.

The fact that the women of this region were chosen to feed the future Elizabeth II was related to the development of transport (such as the railroad) and to their physical condition. Since they were strong and healthy, in constant contact with nature and according to the feminine ideology of the time from the perspective of the predominant functional unit [18,25,34] In this way, the pasiega wet nurses became indispensable for the royal infants, and the wet nurse from Cantabria, mainly from the Pas Valley, became a priority functional framework [25] as a relevant figure in the history of royalty Spanish [10,33]. After the end of the reign of Elizabeth II, the monarchy continued with her son, Alfonso XII. The rearing of infants by wet nurses continued during the reign of Alfonso XII with Mary Christine of Habsburg (Figure 3).

Because of high infant mortality, the government began to promote health. In fact, in the VIII Congress of the General Union of Workers (Madrid) in 1905, a reform of article 9 of the 1900 law was proposed [17,47]. This modification consisted of increasing the period of rest for the working woman after childbirth till six weeks, thus allowing a longer breastfeeding time for the newborn [16,37]. However, due to a lack of knowledge about breastfeeding, as well as the health problems of the biological mother, some women were unable to breastfeed their children. For this reason, in the upper social classes, they hired wet nurses, who could perform this function [19,32,48].

The intercultural differences between rural and urban life made the monarchy choose women dedicated to working in the fields and caring for the family and home [37]. Better physical condition, robustness, as well as wearing loose clothing were considered to favor a better breast condition and, therefore, they could perform their task satisfactorily [41]. This argument contrasted with women in urban areas, where the use of tight clothing, such as the corset, was initiated, which could cause deformation of the chest and difficulties in breastfeeding [30].

### 3.4. Legislation and Regulation of the Job of the Wet Nurse

As a functional unit (UF), the regulations reflected ideology, values, beliefs, customs, and feelings prevailing at that time about the image, the social and labor work of the wet nurse [24,25]. In the Child Protection Act of 1904, which protected children up to ten years of age, Article 8 stated that wet nurses had to deliver a document to the Local Board [14]. It should include marital status, health, conduct, physical conditions, husband’s permission, and birth certificate of the last child. [49,50] This law also requested the inscription in a register of the municipality indicating their health conditions and proof that their children between six and ten months were well fed, in terms of ensuring their survival [14].

Each wet nurse had to have a notebook in which the municipal health inspectors noted the changes of address, endorsed by the mayors [6,51]. This law was later expanded in the RD of 12 April 1910, according to which wet nurses had to have good health and good milk quality, an essential requirement that had been valued since the 19th century to guarantee the survival of newborns [52]. In addition, it regulated the wet nurse hiring agencies, and in this way, they had to write a letter provided by the Local Board [39]. This writing had to be certified by the Mayor, a priest, a doctor, and a judge. Even the director of the agency had to certify good morals. This statement was certified by a multidisciplinary team of doctors and teachers [39,52]. Considering that, in rural areas, these agencies did not exist, the contact between the families and these women was made through “word of mouth” between neighbors, teachers, health professionals and the church [39,53].

### 3.5. Wet Nurse’s Costume: A Social Expression of Wealth

The esthetics of the wet nurses expressed the rendering in the practice of the prevailing ideology on them, constituting the functional unit (UF). They determine the sexual and gender division of work, as well as a labor expression of power. Fact by which, the aspect of the wet nurse should be in line with the idea that had been socially built on it [24,25,54]. The *pasiego* suit underwent changes adjusting to fashion. At first, it was characterized by being dark tones with a bodice and an apron. The open jacket was designed with silk velvet and black satin; it was short and fitted with gold braid and buttons. The skirts were made of black velvet and reached to the height of the ankle. The blouses were made of linen, which in the neck and cuff areas were accompanied by lace [30,46]. The color white was a symbol of social status, so this color in the garments replaced the dark ones, a question that involved an investment of money and time to keep it in good condition [30]. They wore luxurious silver, gold and coral jewelry, which were given as gifts by the family, especially when the child who was breastfed lost a milk tooth. This fact indicated that the infant was well fed and that the wet nurse was fulfilling her function [30,48].

## 4. Discussion

History must be considered as the only science, which is both dynamic and global. This offers us a synthetic vision of the human phenomenon. Therefore, it can be stated that it allows the study of care over time in different societies. This research highlights the rise of wet nurses in the Spanish monarchy. It has identified the social, political, economic, and religious factors which influenced the care provided by these women through breastfeeding in the historical period of 1850–1910 [54]. Therefore, the importance that this figure assumed in relation to the promotion of child health is evident since it facilitated an increase in the birth rate at the end of the 19th and early 20th centuries. This allowed biological mothers to have children in shorter intervals of time. This is a necessary issue due to the high infant mortality rate existing in the study period [10]. Although it is true that in Spain, the birth rates in this period were low, in addition to the existing high infant mortality, as a consequence of the socio-political crisis after the loss of Cuba, the Philippines and Puerto Rico in 1898 [55].

Therefore, at the health level, there was great concern about infant feeding. Breastfeeding was promoted by the government since it increased the survival of infants [56,57]. It should be noted that laws regulated the wet nurses working conditions. These women offered their services in various places and in different social strata. This situation led to the promotion of the establishment of maternity homes, where breastfeeding was provided to children from the most disadvantaged social classes. In addition, the wet nurse card appeared, certifying good physical health, as well as adequate morality of these women [10].

In this study, period (1850–1910), the other worrying issue was related to the health problems of the biological mother (e.g., insufficient milk output, a malformation in the nipple, mastitis) [50]. As Martínez Sabater indicates, the biological roles of women were directed to breastfeeding activities, as a functional element within the framework of the home. This produced a transformation of these tasks, passing them to the wet nurse [2,58]. In addition, at this time, some children did not have a mother, either due to death in childbirth or because they have been abandoned in charities. These were fed through wet nurses [10]. Only as a last recourse, animal milk was used because it was supposed a danger for the newborn due to the increase of the mortality from infections in these children. Although it is true that, during the nineteenth century, in the summer period, there was even 75 percent of deaths of infants due to an increase in gastrointestinal problems [59,60].

The figure of the wet nurse became a privilege among the wealthiest social classes, including the monarchy. The reason for a mother to decide to have her child breastfed by a wet nurse was no longer just a matter of health, but also a preference. Women from the upper social class chose to invest their breastfeeding time in different activities. It is worth highlighting the dedication to esthetic issues and an increase in offspring [10]. Another reason why they delegated breastfeeding to wet nurses was the thought that this practice shortened the life of the mother and worsened her health [42]. It was even noted that husbands of the high social class did not want sexual abstinence and hired wet nurses due to the myth that practicing sexual relations produced irritation in the genitals and even worsened the quality of the milk [44].

The most valued wet nurses were those from Cantabria. For this reason, it was the pasiego costume that became a sign of identity, with the work uniform becoming a symbol of wealth, especially for those who worked for the royalty. It is true that this meant that many women pretended to be pasiegas wearing the costume [30]. In addition, the wet nurses of the monarchy acquired the privilege of being present at public court ceremonies. Their services did not end when the wet-nursing period ended but continued over time, and they acquired the title of *hidalgia* for her and her entire family [44]. For this reason, the pasiega wet nurses wore ostentatious clothing while they strolled through cities such as Madrid, Barcelona, Santander, and San Sebastián. They wore luxurious jewelry given to them by the family, especially when the child had a baby tooth. It is known that to this clothing were added accessories such as gloves, socks, cuffs, all of them made of white cotton [30,61].

The royal wet nurses allowed the survival of those who would be the future kings of Spain in the 19th and 20th centuries. For this reason, the research of the best wet nurse was extensively spread across northern Spain. Due to the strict conditions demanded by the Spanish royalty, several women were chosen to later go back to their place of origin [10,33]. Such is the case of the Santander women Tomasa Antonia López and Brigida Cayón Miranda, brought by the surgeon Solís, who was returned for not meeting the requirements. However, it is shown that the fact of being selected to pass the examinations was a privilege since even if they returned home, they received a salary. In the case of these two women, they were paid 718 reais, as well as the return ticket. After, they were offered compensation for a value of 10.000 reais. This fact shows respect, but also the demand on the part of the court towards these northern women [33,59].

Some women were even more privileged due to their physical and moral conditions, such as the case of Francisca Ramón de Peñacastillo, Isabel II’s wet nurse. It is worth highlighting María Gómez, Alfonso XII’s wet nurse [62,63,64]. Moreover, it is true that during the first half of the nineteenth and early twentieth centuries, infant mortality rates in Western countries were 30 to 60 times higher than today, resulting in the death of five or six children per 1000 live births per year [65,66]. In a period when infant mortality was prevalent, these infants were able to survive thanks to the care of these women. Therefore, a question remains: what would have happened to the history of Spain without the occupation of these women? Would these infants have survived? If not, would the history of Spain be very different?

## 5. Conclusions

This article reveals the importance of the palace wet nurse in the Spanish monarchy in a specific period (1850–1910). These women contributed to increased child survival rates through breastfeeding, as well as other forms of care they provided for their employers’ children. In addition, the economic situation of the families of the wet nurses improved because even the closest relatives were given paid jobs that sometimes facilitated a better future. These activities were carried out both in the city and in the country. These women also had a financial pension after retiring from their profession.

Pasiegas wet nurses are the consequence of a socially constructed cultural ideology (functional unit), which had to be in tune with a geographical context where freshness and health were thought as abundant (functional framework) and, likewise, reflect on their own physiological characteristics and moral.

These women provided an adequate diet to infants during an unstable socioeconomic era, where most of the population survived from work in the fields and in the factories, with terrible wages and in deplorable social and sanitary conditions. Pasiega wet nurses made possible the creation of new family bonds between children and infants, not blood ones, but as “milk brothers”, having been both fed from the same breast. These situations even led to promote this circumstance for years.

Wet nurses lived mainly in the Valle del Pas, although other origins are known, such as Asturias, Burgos, La-Mancha, as well as other areas of Cantabria. This geographical area made possible to be in direct contact with nature for these women, as well as being well nourished and dedicated to working in the fields, caring for the family. Therefore, their choice led the creation of new access routes to the villages where they lived, promoting the trade of food products, livestock and the mobility of people.

The importance of these women happened to be the engine of their “blood” family, directly and indirectly. They were present at royal meetings, celebrations, and some of them even resided in the palace during all her life. A highlight was the clothes of these women as a symbol of that power and wealth that they acquired, once came to be considered as one more member of the family. The clothing was luxurious skirts, blouses, jackets, as well as jewelry.

The wet nurse began to disappear at the end of World War II, at the same time, when the bottle feeding and artificial milk took over. Among other things, this was due to the incorporation of women into the world of work, which promoted the establishment of artificial milk. In addition, industries that promoted infant formulas began to emerge, and it even began to value artificial breastfeeding socially, largely as a fad in the more affluent social classes. In the middle of the twentieth century, wet nurses began to disappear as a profession, transforming feeding into an unpaid activity. Because of the microbial revolution, formula milk gains momentum, leaving breastfeeding to be relegated. With this transformation in the way of raising children, a social debate began considering which alternative was the most suitable for feeding children: breastfeeding or artificial milk.

It is specified that, in some developing countries, there is a current figure of a wet nurse who breastfeeds newborns with health problems. Altruistic breastfeeding continues to exist in tribes of Africa, especially in the case of a mother’s death or illness. It can be associated with the role of the wet nurses with the “milk banks” that exist today. Therefore, we can consider nursing work as one of the first jobs done by women. A physiological issue, such as breastfeeding, has become a way to improve the lives of these families through salary. This event was harshly criticized by society at the time, for the transformation of intimate activity, such as breastfeeding, into a trade. However, the role of these women should be honored and highlighted since they allowed adequate nutrition in a historical period when infant mortality was high.

This article supports the scientific evidence of the advantages of breastfeeding over infant formula. It was due to wet nurses and their breastfeeding that child survival was possible during the XIX and XX centuries. This study specifically approaches the prevention of infant deaths, as well as mortality due to poor nutrition. However, it is true that in some geographical areas figure of the wet nurse has not yet disappeared. The return of wet nurses in Western society is highly likely to be controversial. Nevertheless, this article emphasizes the importance of health policies to promote breastfeeding through health figures such as specialist nurses in obstetrics and gynecology, as well as pediatric nurses.

The relevance of this study is justified by three fundamental reasons, on one hand, in view of the need to know the incidence of royal wet nurses in the reduction of infant mortality in Spain (not only in the court environment, but also in that sector of society which, imitating this practice, incorporates it into its daily life), and second, by the importance of the influence of monarchs and their environment—especially high courts—in the selection and promotion of wet nurses inside and outside the court. Finally, this review can serve as a reference in the Hispanic context, to carry out comparative studies analyzing the similarities and differences that have existed between the royal wet nurses of the different European monarchies at the time studied and to verify their relationship with the existence of breastfeeding and mercenary patterns in Europe at the time.

## Figures and Tables

**Table 1:** Wet nurse industry legislation.

Legislation	Period	Observations
Royal Jurisdiction of Castilla	1252–1255	Abandonment of children was punished. A person who let a child die for not feeding him was sentenced to death.
“Partidas” “Novísima Recopilación”	1805	They reported on how to raise children. Prevents the way to proceed in cases of orphanhood.
General Benefit Regulations	27 December 1821 Art. 58–61	Opening of a maternity home for breastfeeding. Two work periods for the wet nurses are established: two years of lactation and subsequent raising.
Law of 23 January 1822	Title III. Arts.41 y 50–70	Regulation of the living conditions of orphaned children in Charity establishments.
Law of 20 June 1848	1848	Protection of foundlings, orphans and the homeless
Regulations of 9 March 1862	1862	General conditions that must have the external wet nurses in Alicante.
Law of 12 August 1904	1904	It extended its protection to all children under the age of ten, and the Higher Council for Children with Provincial and Local Boards was created as a regulatory body.
Regulation 24 January 1908	1908	
Regulation 24 January 1908	1908	

Source: authors’ own elaboration from historical laws and Spanish Gazette: historical collection from 1661 to 1959.

**Table 2:** Thematics blocks related to the references.

Database	Search Strategy	Limits	Points Extracted	Reference
Pubmed Cochrane CINHAL Scopus Google Scholar Books	breastfeeding AND human milk human lactation AND breastfeeding breastfeeding OR human milk breastfeeding AND human lactation infant mortality AND child survival Intercultural care AND society historical research AND society breastfeeding OR health promotion wet nursing and breastfeeding nurse AND wet nursing infant care OR child survival	Title Article English/Spanish	Origin Start of the selection process Privileges of the royal wet nurses	[28,29,30,31,32,33,34,35,36]
			Wet nurse selection process Wet nurse’s salary	[37,38,39,40,41,42,43,44,45,46]
			Origin of the monarchical wet nurse	[47,48]
			Legislation	[49,50,51,52,53]
			Wet nurse’s costume (wealth)	[54]

Source: authors’ own elaboration.

**Table 3:** Documentation encompassed within the socio-health reality in Spain in relation to the mother of the royalty.

Author(s)	Type of Document/Study Year	Purpose	Characteristics	Results
Siles-Gonzalez et al. [24]	Book 2016	Educational Anthropology of Care	Ethnography and ethnology Context: interpretative paradigm	Culture Customs Society
Barona, J.L. et al. [28]	Book 2008	Spanish society’s health	Start of the selection process	Nutrition Infant feeding Breastfeeding
Gustavo, C. [30]	Book 1999	The costume in Cantabria (Santander)	Pasiega wet nurse’s clothes	White color: wealth Different dress: urban/rural area Gifts Jewelry: good work Added: white clothing accessories
Gacho Santamaría, M.A. [31]	Journal (1995)	Doctors and wet nurses of the Spanish Court (1625–1830)	Names of physician-surgeons and wet nursing	Start of the selection process
Gil, J.M.F. [32]	Journal (1995)	Wet nurses Peasant women in the city	The upper social classes hired wet nurses to breastfeed the infant	Privileges of the royal wet nurses
Cortés Echanove. [33]	Book (1958)	Birth and upbringing of real people in the court of Spain	Contact of the monarchy with the town councils and districts, which oversaw notifying the future wet nurses	Women with more than one child; Important figure of Spanish royalty
Buldain, B. [34]	Book (1958)	Contemporary History of Spain, 1808–1923	Good family health (woman, man, children)	Social perspective era: predominant functional unit
Espasa, H.d.J. [35]	Book (1958)	Universal Illustrated European-American Encyclopedia	Amount of hair Skin infections Digestive disorders Appropriate age of wet nurses	Anatomical and physical features a wet nurse
A.C.d. [36]	Notebook (1889)	Wet nurse’s notebook	Notebook, where the contracts and dismissals of the different families in which they worked were sealed	Possession of the notebook was mandatory for performing wet nurse’s work
Aguilar Cordero, M. [37]	Book (2005)	Breastfeeding	Ruggedness, loose clothes	Assessment test: vigorous, no breathing problems
Iberti, J. [38]	Manual (1789)	Artificial method of raising newborn children and giving them a good physical education	Associated features witchcraft	First moments attention child; Breeding mothers Exercise of the wet nurse
Siles, J. et al. [39]	Journal (1998)	The biological link in the history of health care, the case of wet nurses: An anthropological view of nursing	Arrival: medical contingency for oral transmission: priest, neighbors; Official mother’s document Official Certification	Number of the wet-nurse’s children with whom she lived, when her last child was born, her work, and her husband’s one, vaccination card, proof of good health, milk analysis, and good morals
Junceda Avello, E. [40]	Book (1995)	Gynecology and intimate life of the queens of Spain	Checking the quality of the milk Quota for finding a wet nurse	Rich in sugars, fats- Milk quality assessment equipment
Toquero Sandoval, C. [41]	Manual (1617)	Rules for choosing wet nurses and milk	Wet nurses with at least two children The children must breastfeed for at least two months	Exclusion for defects
Zabía Lasala, M.P. [42]	Dictionary (1999)	Dictionary of Juan Alonso and the Ruyzes de Fontecha	Milk quality testing equipment Reasons to hire a wet nurse	Scales of the time; Delegating breastfeeding to wet nurses; It was believed that breastfeeding shortened the life of the biological mother and worsened her health
Amezcua, M. [43]	Journal (2000)	The esthetics of the wet nurses	Wet nurse selection process Wet nurse’s salary	Difficulties of the first-time wet nurse: poorer quality milk
Sarasúa, C. [44]	Journal (1994)	Domestic service in the formation of the Madrid job market, 1758–1868	Economic retribution and lifetime pay	Christian morality, values and correct customs
Fildes, V. [45]	Book (1986)	Breasts, bottles, and babies—a history of infant feeding	Wet nurse selection process Wet nurse’s salary	Improvement of the economic situation of the family
Pérez, T.G. [46]	Journal (2012)	Study of the role of the pasiega wet nurse in Spanish in the 19th and early 20th centuries	Symbology of clothing Sociocultural changes	Black and white dress, wet nurse; Improves the health of real infants Increased survival of wet nurse’s children
Castells, I. [47]	Book (2003)	Origins of liberalism; University, politics, economy	Origin of the monarchical wet nurse	Political reforms; Health Promotion
Soler, E. [48]	Book (2010)	Milk brothers and social mobility; The pasiega wet nurse; Families	Refusal to breastfeed by biological mother Relationship: rural/urban woman	Lack of knowledge about breastfeeding Gifts to wet nurse for milk quality: Objectivity of good breastfeeding: teething
Orzes, M.d.C.C. [50]	Journal (2007)	Nurses and mercenary breastfeeding in Spain during the first third of the 20th century	Health problems of the biological mother	Medical disorders (mastitis)
Salazar-Agulló, M. [51]	Book (2009)	Mother and childcare and gender issues in the program “At the Service of Spain and the Spanish Child” (1938–1963)	Individual wet nurse’s notebook reviewed by inspector	Change of address
Scott, J. [52]	Journal (1993)	The working woman in the 19th century; Women’s History 1993	Wet nurses’ agency: good health good milk quality,	Essential requirements for hiring
Martín, A.M.R. [53]	Journal (2008)	The destiny of the children of the orphanage of Pontevedra, 1872–1903	Nurse hiring management: multidisciplinary group	Mayor, pastor, Doctor

Source: Own elaboration of the authors.

**Table 4:** Parenting of Elizabeth II’s children by wet nurses from northern Spain.

Date of Birth	Name of the Children	Physician	Wet Nurse	Husband	Place of Origin
12 July 1850	Male: dies at birth due to childbirth problems	Pedro Castelló Marqués José Figuer y Cubero Doctor Pedro Gilly	Francisca Guadalupe María Pelayo Agustina de Larrañaga y Olave	Miguel González de Villegas Pedro Herrero Juan Bautista de Zabaleta	Valle de Toranzo (Santander)
20 December 1851	Mª Isabel Francisca de Asís	Jaime Drumen Dionisio Solís Francisco Alonso Rubio Francisco Alarcos	María Sabatés de Plavevall (retén) Nobody was chosen Cecilia Pastor (wet nurse chosen) Vicenta Valenciaga (2nd wet nurse)	Unknown — Facundo Montes Teodoro Celada	Vich (Barcelona) Santander, Navarra y Zaragoza Turégano (Segovia) Vasongadas
5 January 1854	Infanta Cristina (she died on 7 January 1854)	Alonso Rubio	Celestina de Diego (urgent choice for breastfeeding problem)	Dionisio Gómez	Valle del Pas
11 November 1857	Alfonso XII	Francisco Alonso Rubio Francisco Antoño Alarcós	María Gómez Josefa Ruíz Oria (2nd wet nurse)	Juan Mantecón Antonio Ruiz Navedas	Valle del Pas Valle del Pas
26 December 1859	Mª Concepción Francisca of Asís (she died before 2 years old)	Francisco Alonso Rubio Francisco Antoño Alarcós	Manuela Oria Ruiz Petra Arroyo (2nd wet nurse)	Agustín Gómez Ambrosio Vivar	Santander Burgos
4 June 1861	Mª Pilar Berengüela	Don Bruno Agüera	Juliana Revilla Araus (wet nurse chosen) Úrsula Leonor (2nd wet nurse) Manuela Cobo (wet nurse chosen)	Víctor Revilla González Unknown Unknown	Villamayor de los Montes (Burgos) Cabañas de Juarros (Burgos) San Roque de Rio Miera (Santander)
23 June 1862	Infanta Doña María de la Paz	Don Bruno Agüera	Cecilia García (2nd wet nurse) Andrea Aragón (wet nurse chosen)	Unknown Unknown	Cabañas de Juarros (Burgos) Carazo (Burgos)
12 February 1864	Doña Eulalia de Borbón	Don Manuel Izquierda	Lorenza García (2nd wet nurse)	Tomás Alonso	Carcero de Bureda (Burgos)
14 February 1866	Francisco de Asís Leopoldo (died within hours of birth)	-	-	-	-

Source: own elaboration from the authors. Adapted from [33].

## Abbreviations

Dialectical structural model of care	DSMC
Functional unit	UF
Functional framework	MF
Functional element	EF
